# The problem with dichotomizing quality improvement measures

**DOI:** 10.1186/s12871-022-01833-z

**Published:** 2022-09-19

**Authors:** James Harvey Jones, Neal Fleming

**Affiliations:** 1 Department of Anesthesiology, University of North Carolina at Chapel Hill, N2198 UNC Hospitals, CB #7010, Chapel Hill, NC 27599-7010 USA; 2 Department of Anesthesiology and Pain Medicine, University of California Davis Medical Center, 4150 V Street, PSSB Suite 1200, Sacramento, CA 95817 USA

**Keywords:** Quality improvement, Statistical process control, G chart, P chart

## Abstract

The Anesthesia Quality Institute (AQI) promotes improvements in clinical care outcomes by managing data entered in the National Anesthesia Clinical Outcomes Registry (NACOR). Each case included in NACOR is classified as “performance met” or “performance not met” and expressed as a percentage for a length of time. The clarity associated with this binary classification is associated with limitations on data analysis and presentations that may not be optimal guides to evaluate the quality of care. High compliance benchmarks present another obstacle for evaluating quality. Traditional approaches for interpreting statistical process control (SPC) charts depend on data points above and below a center line, which may not provide adequate characterizations of a QI process with a low failure rate, or few possible data points below the center line. This article demonstrates the limitations associated with the use of binary datasets to evaluate the quality of care at an individual organization with QI measures, describes a method for characterizing binary data with continuous variables and presents a solution to analyze rare QI events using g charts.

**Supplementary Information:**

The online version contains supplementary material available at 10.1186/s12871-022-01833-z.

## Background

The Anesthesia Quality Institute (AQI) promotes improvements in clinical care outcomes by managing data entered in the National Anesthesia Clinical Outcomes Registry (NACOR) [1]. Anesthesia departments utilize NACOR reports to evaluate their overall and individual performances with respect to quality metrics as compared to national benchmarks and guide modifications of clinical practices. Each case included in NACOR is classified as “performance met” or “performance not met” and expressed as a percentage for a length of time: “performance met” over total included cases per month.

The clarity associated with this binary classification presents limitations on data analysis that may not be optimal guides to evaluate the quality of care at an individual organization. Binary data (met/not met) preclude statistical characterizations of distribution parameters, such as the mean and variability [2]. Rephrasing clinical questions to include continuous variables may better guide continuous quality improvement. Prior investigators highlighted this strategy with respect to the rate of surgical site infections (SSIs) [3]. Rather than a binary dataset consisting of attribute variables (“infection” or “no infection”), a more informative dataset incorporates the number of cases between consecutive SSIs [3]. The problems associated with dichotomization of continuous variables have been highlighted with respect to clinical research in anesthesia [4]. We present the consequences of dichotomization within the context of current AQI metrics and their utility for evaluating quality at an individual organization.

High compliance benchmarks present another obstacle for evaluating performances. In 2020, AQI 63 (Neuromuscular Blockade: Documented Assessment of Neuromuscular Function Prior to Extubation) was rejected by the Centers for Medicare and Medicaid Services (CMS) for reporting as a Qualified Clinical Data Registry (QCDR) measure due to its high performance rate and lack of variability. Traditional approaches for interpreting statistical process control (SPC) charts depend on data points above and below a center line, which may not provide adequate characterizations of a QI process with a low failure rate, or few possible data points below the center line [5, 6]. This article demonstrates the limitations associated with the use of binary datasets to evaluate performance with AQI measures, describes a method for characterizing binary data with continuous variables and presents a solution to analyze rare QI events using g charts.

### Binary versus continuous data

There are two main data types that will be discussed within the context of evaluating quality of care: binary (also known as attribute) and continuous (also known as variable). For QI measures, binary datapoints are often expressed as “performance met” or “performance not met.” Therefore, the percent compliance can be described with the following expression:

$${}^{"} performance\ {met}^{"}/\left({}^{"} performance\ {met}^{"}+{}^{"} performance\ not\ {met}^{"}\right)$$

The assessment of neuromuscular function prior to extubation was given high priority status as an internal improvement measure (IIM) in the NACOR and will serve as an example for our discussion [7]. Residual neuromuscular blockade may lead to postoperative respiratory complications and it is reasonable to assume that the relative risk of complications increases with the magnitude of the residual blockade [8]. The train-of-four (TOF) ratio is used to assess neuromuscular function and satisfies this measure with decimal fraction values ranging from 0 to 1.0. A TOF ratio greater than 0.9 indicates resolution of neuromuscular blockade. A TOF ratio less than 0.9 indicates residual neuromuscular blockade. Dichotomization of TOF ratios to “performance met” or “performance not met” sacrifices data regarding the distribution of neuromuscular function for patients as well as its intraoperative assessment by anesthesiologists. In contrast, continuous data have infinite numerical values and can yield the mean and variability for a dataset as well as information regarding the performance of the QI process relative to control limits.

### Limitations of binary data

Binary data analyses are limited to comparisons between populations or between a population and a target value. For example, the two-sample t-test and the two-group test to compare variances can characterize the performances of two individual providers or departments relative to each other or to a benchmark. As the comparative populations become more similar or performances approach the benchmark, greater sample sizes are needed to detect statistically significant differences. Binary data cannot provide critical distribution parameters, such as mean or standard deviation. Compliance can only be expressed as percentages for a specified time.

### Expressing binary data with continuous variables

Alternatively, it is possible to express compliance with QI measures as the number of cases recorded before a case fails to meet the compliance criteria, thus creating a continuous dataset. For example: the NACOR benchmark for a given measure is 97.58% compliance. Expressed another way, 2.42% of cases (2.42 cases per 100) can be defective (“performance not met”) and still satisfy the benchmark. The following expression can be used to determine the minimum number of cases between consecutive noncompliant cases, where X = # defects / month (where defect = case recorded as “performance not met”):

$$\left(\raisebox{1ex}{$1$}\!\left/ \!\raisebox{-1ex}{$X$}\right.\right)\times \left(\# cases/ month\right)=\# cases/ defect$$

This expression can be further simplified:

$$\left(\# cases/ month\right)/X=\# cases/ defect$$

Analyzing the number of cases between consecutive defects still employs dichotomization of neuromuscular function to either “performance met” or “performance not met.” However, improved characterization of these data can be accomplished with the continuous dataset.

### Advantages of continuous data

Continuous data allow for distribution analyses, including mean and variability of a process. Consider the following example. Two anesthesiologists (A and B) demonstrate identical compliance (97.5%) with a QI measure and complete 100 cases each month. Before one case fails compliance, anesthesiologist A and B each complete an average of 40 cases. Although the two anesthesiologists demonstrate similar compliances, there can be differences among the distribution of cases that fail compliance. Knowledge of dataset distribution parameters can better guide the QI processes, especially in conjunction with the appropriate SPC chart.

### Statistical process control (SPC) and variation

Choosing the appropriate SPC chart for the dataset is critical to recognize common cause and special cause variations. SPC chart selection should first consider the outcome of interest. While some projects may only be interested in whether a case has met or failed compliance (also known as defective), other projects may be focused on reducing the number of defects, which may or may not reduce the total number of defective cases. For example, a defective case (such as residual postoperative neuromuscular blockade) may contain one or more defects (failure to monitor TOF count, failure to administer appropriate reversal drug, or inappropriate reversal dose, etc.). However, a defect (such as failure to monitor TOF count) may not always lead to a defective case (such as residual postoperative neuromuscular blockade). While p and np charts are most appropriate for detecting the proportion of defective cases within a sample, c and u charts are most appropriate for detecting the number of defects within a sample. Sample size is also an important factor when selecting an SPC chart. While p and u charts evaluate the quality of care with varying sample sizes, np and c charts require a constant sample size.

**Fig. 1:**
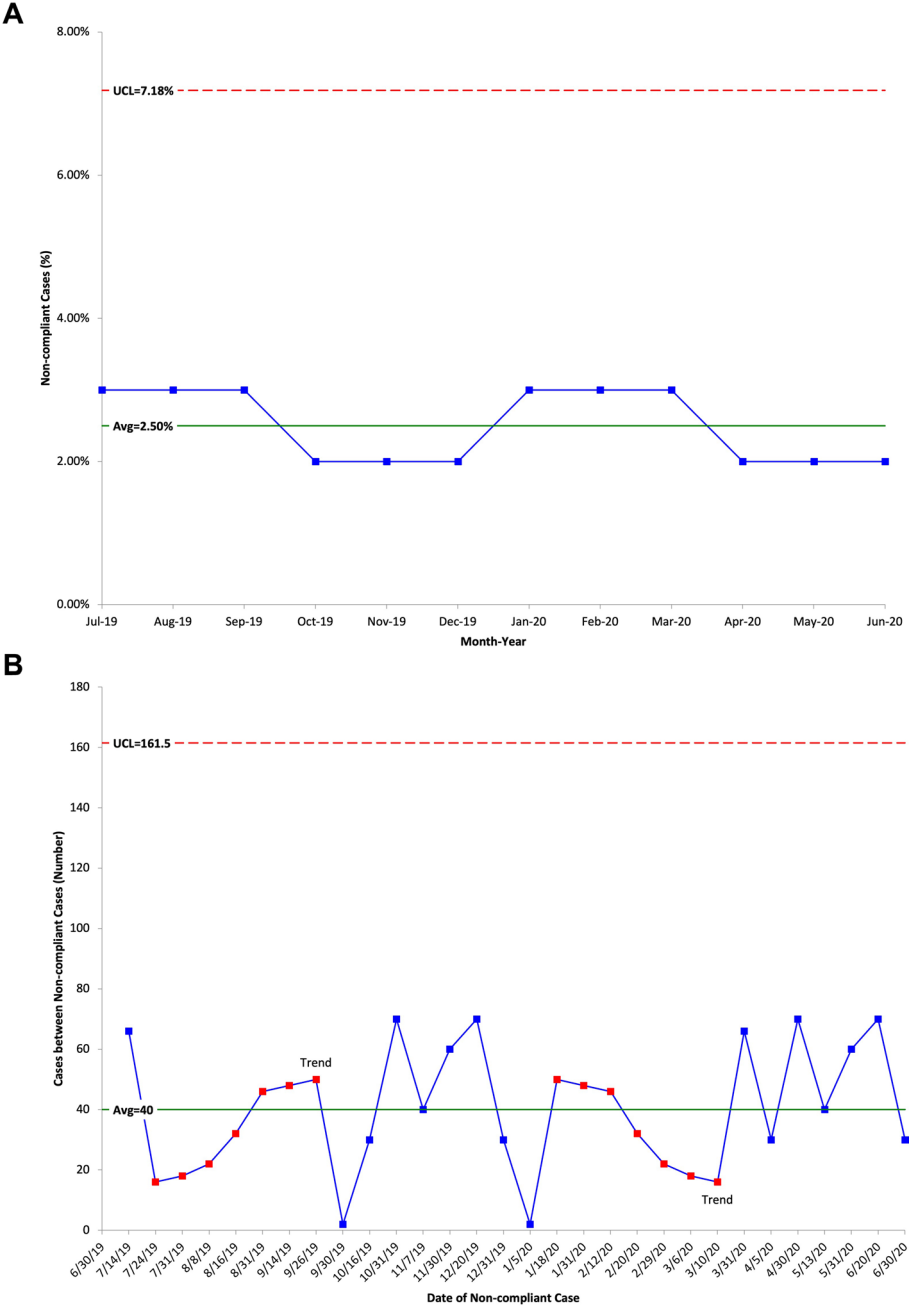
**A** P chart for percentage of non-compliant cases from July 2019 through June 2020. **B** G chart for number of cases between non-compliant cases from July 2019 to July 2020; opposite trends are shown in red from 7/24/19–9/26/19 and from 1/18/20–3/10/20. UCL = Upper Control Limit. Avg = Average

The high benchmark compliance (> 97%) for most AQI measures implies that the average number of defects over time may be very small. Commonly used SPC charts (np, p, u, and c) for attribute data may not clearly demonstrate process improvements if defects are rare. Figure 1A shows a p chart for an anesthesiologist with 97.5% compliance and an average of 100 cases per month. Given the low average monthly defects, this chart would not provide timely feedback to anesthesiologists seeking process improvement. At least 7 months of near-perfect compliance must elapse before judging the impact of an intervention.

Prior researchers have investigated the performances of different time series charts to detect process changes with binary data. A narrative review on this topic determined that g charts are helpful for monitoring rare adverse events [6]. The authors also concluded that cumulative sum (CUSUM) charts may be the most efficient method for detecting “small absolute increases in rates of less than 10%” compared to the Shewhart p-chart and exponentially weighted moving average (EWMA) chart, yet noted that CUSUM charts are difficult to generate and interpret [6].

### Advantages of g charts

G charts display changes over time in the number of anesthetic cases performed between consecutive noncompliant cases. Unlike data for p charts, data for g charts do not reset at arbitrary timepoints (monthly, for example). Therefore, g charts may detect changes in QI performance more quickly. Figure 1B shows a g chart for the number of anesthetic cases performed between consecutive defects (cases that fail to meet compliance criteria) and an overall compliance rate of 97.5% over a one-year period. Although both charts are created from the same dataset, special cause variation in the form of trends are only appreciated with the g chart.

Review of the literature regarding G charts was performed using a basic search in PubMed. The search query of “G chart” yielded 14 results, yet only one publication was identified as pertinent to the current manuscript [9]. The authors of this Norwegian study employed statistical process control with G chart to evaluate the number of caesarian surgeries between infections and concluded that the “result was demonstrated elegantly as a marked shift in process in g-chart…[and] found the g-chart was efficient, sensitive and simple to handle” [9].

Because of the limited number of relevant search results, the PubMed query was expanded to include the following terms: (“binary” OR “binomial” OR “Bernoulli”) AND (“control chart” OR “time series” OR “shewhart” OR “cusum” OR “cumulative sum” OR “g chart”) AND (anesthesiology OR anesthesia OR perioperative). The title and/or abstract of all ten studies were reviewed for their relevance to perioperative patient care and anesthesia-related quality improvement. Publications were not included for the following reasons: not pertinent to quality improvement research (one article), not relevant to perioperative care (three articles) and primary focus on statistical methods (one article). Five articles were identified as pertinent to the current manuscript. One article narrates a department’s experiences with CUSUM control charts for monitoring anesthesiologists’ performance ratings by trainees and nurse anesthetists [10]. The authors’ conclusions highlight the assumptions, and subsequent limitations, of using CUSUM charts for perioperative research and served as practical limitations for the study: the outcome is assumed to be binary; there is immediate feedback regarding performance; each observation is statistically independent; and each outcome has an equal probability of being below the binary cutoff [10]. Despite these limitations, CUSUM charts promptly detected low supervision performance scores [10]. Another article analyzed physician staffing utilization during on-call hours and found that Shewhart charts perform comparably with CUSUM charts [11]. A third article demonstrated that surgeons’ monitoring of time (days) to cholecystectomy increased the rates of early cholecystectomy for patients requiring admission [12]. CUSUM charts were used effectively to monitor sequential outcomes following coronary artery bypass surgery [13]. Last, CUSUM analyses identified the minimum time required for EMLA cream to be effective [14]. This more comprehensive literature review shows the range of applications for CUSUM charts, how they perform relative to traditional SPC charts and also a gap in the literature where g charts may have provided benefit. Discussions within individual departments to use traditional SPC charts, CUSUM charts, or g charts for analyzing QI datasets should include the frequency of data collection and analysis; the baseline performance rate of the process measure; and how the baseline performance compares to the goal performance rate, or national standards.

## Conclusion

There are significant limitations with dichotomized QI data leading to the inability to evaluate clinical practices in a timely manner, particularly for high-performing QI measures. Although the simplicity of binary datasets allows for quick assessments of progress, for clinicians with limited formal education in medical statistics, re-characterizing these same data with continuous variables may lead to more actionable process evaluations. Future QI initiatives should consider the impact of dichotomization on data interpretation and presentation. Electronic compliance dashboards that display continuous data with g charts may allow for immediate recognition of special cause variations and prompt corrective action(s).

In summary, this manuscript highlights the following points related to the evaluation of quality of care at an individual organization with respect to AQI measures:

- Binary datasets are associated with limitations on data analysis and presentations that may not provide optimal guides to improve clinical care.
- Continuous datasets consisting of the number of cases between consecutive non-compliant cases allow for more informative statistical analyses.
- Special cause variation for high performing QI measures may require continuous datasets and analyses with g charts.
- Methods for characterizing binary data with continuous variables are critical for guiding continuous QI initiatives at individual organizations.

## Supplementary Information

**Additional file 1.**

## Data Availability

All hypothetical data used in this article to create the figures are included in its supplementary information files.
